# Application value of intraoperative electrophysiological monitoring in cerebral eloquent area glioma surgery: a retrospective cohort study

**DOI:** 10.1007/s12672-024-00975-5

**Published:** 2024-04-13

**Authors:** Yuankun Liu, Songyun Zhao, Jin Huang, Pengpeng Zhang, Qi Wang, Zhuwen Chen, Lingjie Zhu, Wei Ji, Chao Cheng

**Affiliations:** 1 Department of Neurosurgery, The Affiliated Wuxi People’s Hospital of Nanjing Medical University, Wuxi, China; 2 Wuxi Medical Center, Nanjing Medical University, Wuxi, China; 3 Department of Lung Cancer Surgery, Tianjin Medical University Cancer Institute and Hospital, Tianjin, China; 4 Department of Gastroenterology, Affiliated Hospital of Jiangsu University, Zhenjiang, China; 5 Department of Functional Neurosurgery, The Affiliated Wuxi People’s Hospital of Nanjing Medical University, Wuxi, China; 6 Department of Pathophysiology, School of Medicine, Nantong University, Nantong, China

**Keywords:** Glioma, Intraoperative neurophysiological monitoring, Function, Survival, Retrospective analysis

## Abstract

**Introduction:**

Surgery for gliomas involving eloquent areas is a very challenging microsurgical procedure. Maximizing both the extent of resection (EOR) and preservation of neurological function have always been the focus of attention. Intraoperative neurophysiological monitoring (IONM) is widely used in this kind of surgery. The purpose of this study was to evaluate the efficacy of IONM in eloquent area glioma surgery.

**Methods:**

Sixty-eight glioma patients who underwent surgical treatment from 2014 to 2019 were included in this retrospective cohort study, which focused on eloquent areas. Clinical indicators and IONM data were analysed preoperatively, two weeks after surgery, and at the final follow-up. Logistic regression, Cox regression, and Kaplan‒Meier analyses were performed, and nomograms were then established for predicting prognosis. The diagnostic value of the IONM indicator was evaluated by the receiver operating characteristic (ROC) curve.

**Results:**

IONM had no effect on the postoperative outcomes, including EOR, intraoperative bleeding volume, duration of surgery, length of hospital stay, and neurological function status. However, at the three-month follow-up, the percentage of patients who had deteriorated function in the monitored group was significantly lower than that in the unmonitored group (23.3% vs. 52.6%; P < 0.05). Logistic regression analysis showed that IONM was a significant factor in long-term neurological function (OR = 0.23, 95% CI (0.07–0.70). In the survival analysis, long-term neurological deterioration indicated worsened overall survival (OS) and progression-free survival (PFS). A prognostic nomogram was established through Cox regression model analysis, which could predict the probability 3-year survival rate. The concordance index was 0.761 (95% CI 0.734–0.788). The sensitivity and specificity of IONM evoked potential (SSEP and TCeMEP) were 0.875 and 0.909, respectively. In the ROC curve analysis, the area under the curve (AUC) for the SSEP and TCeMEP curves was 0.892 (P < 0.05).

**Conclusions:**

The application of IONM could improve long-term neurological function, which is closely related to prognosis and can be used as an independent prognostic factor. IONM is practical and widely available for predicting postoperative functional deficits in patients with eloquent area glioma.

**Supplementary Information:**

The online version contains supplementary material available at 10.1007/s12672-024-00975-5.

## Introduction

Malignant glioma involving eloquent areas and subcortical fibres is very difficult to perform complete resection and prone to cause various neurological dysfunctions [1], which seriously affects the quality of life and prognosis of patients [2]. Despite the continuous development of diagnostic and treatment methods in recent years, the clinical effect is still unsatisfactory, and the five-year survival rate is less than 5% worldwide [3]. The surgical strategy for patients with eloquent areas of glioma is to maximize tumour resection while preserving neurological function as much as possible [4]. On the one hand, EOR is positively correlated with OS and considered to be an independent prognostic factor affecting survival, regardless of low grade or high grade glioma [5]. On the other hand, severe postoperative neurological deficits, such as hemiplegia and aphasia, will also affect the quality of life and prognosis of patients [6].

With the progress of preoperative functional imaging and IONM technology, as well as the further understanding of brain functional plasticity [7], many studies have confirmed that IONM combined with preoperative functional magnetic resonance imaging (fMRI), diffusion tensor imaging (DTI), and intraoperative neuronavigation could provide the possibility of achieving a balance between EOR and protection of neurological function [8, 9]. IONM technology started in the 1990s and has been widely used in the past decade [10]. It is a valuable technique for monitoring the function of the nervous system in at-risk conditions, including the surgical operation of deficits in neurological function or physiological changes in the internal environment [8]. Most transient injuries to the nerve, such as prolonged retraction, excessive electrocoagulation and obstructed blood circulation, are easily corrected during surgery [11, 12]. By understanding the changes in electrophysiological signals in the process of nerve transmission, surgeons can judge the integrity of neurological function in a timely and comprehensive manner, improve the quality of decision-making and ultimately reduce the rate of surgical disability [13, 14]].

Since 2016, IONM technology has been introduced in our hospital and widely applied to multiple neurosurgical procedures. However, there is a lack of statistical evidence in our hospital to evaluate the effect of IONM on the protection of neurological function and prognosis in eloquent areas of glioma surgery, as well as the predictive value of various IONM indicators. Therefore, this study retrospectively analysed the clinical application value of multimodal IONM technology in cerebral eloquent area glioma surgery in our neurological disease diagnosis and treatment centre.

## Materials and methods

### Patient selection

This study was approved by the ethics committee of Wuxi People’s Hospital Affiliated to Nanjing Medical University (Jiangsu, China) under approval number KY23086. All surgical patients signed informed consent forms and agreed to provide clinical data for research purposes. The present investigation harnessed data sourced from the clinical databases of Wuxi People's Hospital. The IONM technology was conducted after 2016. Patients were divided into two groups on the basis of the application of tailored IONM (Monitored group, 2016–2019, n = 30) or not (Unmonitored group, 2014–2016, n = 38) between January 2014 and December 2019.

The inclusion criteria were as follows: (1) patients underwent surgery for the first time, and the postoperative pathological specimen was confirmed as glioma; (2) both preoperative and postoperative imaging data were complete, and the tumour was located in the eloquent area, the “eloquent area” of glioma was defined as the sensorimotor area where the tumor involves or is closely adjacent to the dominant hemisphere (inferior frontal gyrus, superior temporal gyrus and inferior parietal lobules) and some subcortical areas (basal ganglia and thalamus); (3) preoperative Karnofsky functional status (KPS) score > 60 and Eastern Cooperative Oncology Group (ECOG) grade < grade II; and (4) clinical data and follow-up data were complete, and the follow-up time was more than 36 months. Exclusion criteria: (1) Patients with other malignant tumours, severe liver and renal dysfunction, severe lung and heart failure, coagulation dysfunction and other autoimmune diseases; (2) The tumour was located in the language area because surgery for language areas gliomas requires intraoperative awakening, which requires more complex surgical and anaesthesia teams.

### Preoperative preparation and anaesthetic procedure

All patients were evaluated by a 3.0T superconducting MR scanner (Philips Medical Systems, Andover, MA, USA) including MRA, MRS, fMRI and DTI before the operation, and digital subtraction angiography (DSA) was also performed if necessary. A neuronavigation system (Stealth Station S7 Surgical Navigation System; Medtronic, Dublin, Ireland) was used to locate tumours. Detailed treatment plans were made by experienced chief physicians.

For IONM anesthesia protocols, We referred to the 2019 American Society of Neurophysiological Monitoring (ASNM) guidelines [15] and the Chinese Expert Consensus on electrophysiological Monitoring and Anesthesia in Neurosurgery (2021). The anaesthesiology department of our hospital adopted intravenous and inhalational combined anaesthesia or total intravenous anaesthesia, without muscle relaxants during the whole monitoring process. The MAC of inhaled anaesthesia was controlled below 0.5 MAC.

### Monitoring method and alarm criteria

A 32-channel Cadwell Cascade Pro (Cadwell Industries Inc., Kennewick, WA, USA) was used to record IONM data during surgical procedures. Monitoring methods included somatosensory evoked potentials (SSEP), transcranial electrical stimulation motor evoked potentials (TCeMEP), cortical somatosensory evoked potentials (SEP), direct corticoelectrical stimulation (DCS), direct subcortical electrical stimulation (DsCS), electromyography (EMG) and cortical electroencephalogram (ECoG).

SSEP can monitor the function of sensory nerve conduction. For tumours near the central sulcus, phase inversion of SEPs can determine the localization of the central sulcus. TCeMEP can monitor the integrity of the corticospinal tract and assess motor function. For tumours involving the precentral gyrus, DCS was used to map the motor area. For resection of deep-located gliomas adjacent to the pyramidal tract, DsCS was performed to determine the relationship between the scope of resection and the motor conduction pathway. Unlike evoked potentials, EMGs record the spontaneous electrical activity of muscle groups innervated by the surgical area. ECoG can reflect the perfusion of the brain and the depth of anaesthesia.

The IONM parameter settings and alarm criteria were based on the 2014 American Society of Neurophysiological Monitoring (ASNM) guidelines, which were updated in 2019 [15].

### Outcome collection and evaluation

Basic patient information was collected from the electronic medical records, and neuroimaging data were obtained from the Picture Archiving and Communication Systems (PACS) of our hospital. EOR was defined according to the Response Assessment in Neuro‐Oncology (RANO) criteria [16]. High-grade gliomas (HGG) were delineated on T1-weighted enhanced MRI images, and lower grade gliomas (LGG) were delineated on T2-weighted FLAIR MRI images. Postoperative MRI was generally performed within 24 to 72 h. OsiriX MD software (Pixmeo SARL, Bernex, Switzerland) was used to process MRI images and determine the preoperative tumour volume (TV) and postoperative residual tumour volume (RV). The EOR is calculated as follows: EOR = (TV−RV)/TV.

The neurological function of patients was evaluated based on the Karnofsky Performance Status (KPS) score and Eastern Cooperative Oncology Group (ECOG) grade, which were calculated preoperatively, two weeks after surgery, and at the three-month follow-up. Then, we examined the neurological function change of each patient at different periods. According to KPS score change and ECOG grade, improvement: KPS score increment ≥ 10 points or ECOG grade decrement ≥ I grade; unchanged: no significant change in KPS score and ECOG grade; deterioration: KPS score decrement ≥ 10 points or ECOG grade increment ≥ I grade. Short-term functional follow-up was defined as two weeks postoperation, while long-term follow-up was defined as three months.

### Follow-up

The follow-up methods included outpatient review, telephone calls and text messages. After completion of the treatment, patients were interviewed every three months for the first three years and every six months thereafter annually. OS was calculated from the starting date of surgery until the date of death or last follow-up. PFS was calculated from the starting date of surgery to the date of the time of progression/recurrence or last follow-up.

### Statistical analysis

The Kolmogorov‒Smirnov test was performed to test the distribution of continuous variables. For normally distributed data, Student’s t test was performed, and the Mann‒Whitney test was used when the criteria for normality were not met. Categorical variables were assessed using chi-square tests. Logistic regression analyses were used to detect the independence of related factors. Survival curves were plotted by using the Kaplan–Meier method and compared using the log-rank test. Survival prognosis was investigated with univariate and multivariate Cox regression models, in which variables with P < 0.10 in univariate analyses were entered into multivariate analyses. Variables with P < 0.05 were considered statistically significant. Statistical evaluation was performed using GraphPad Prism version 8.0 (Graphpad Software Inc., San Diego, CA, USA) and SPSS Statistics version 26.0 (IBM SPSS, Inc., Chicago, IL, USA).

A nomogram was established to predict the 3-year survival rate by using the RMS package of R. The accuracy of predictions was evaluated by estimating the model’s calibration, and discrimination was measured by Harrell’s concordance index (C-index). Calibration curves were assessed graphically by mapping the observed rates against the nomogram-predicted probabilities. Finally, ROC curve analysis was performed using the pROC package and the ggplot2 package for visualization. Related statistical evaluation was conducted using R 3.4.4 software (Institute for Statistics and Mathematics, Vienna, Austria).

## Results

### Demographic characteristics and clinical data of the patients.

A total of 68 cerebral eloquent area glioma patients were identified and divided into two groups according to whether IONM was used: the monitored group (n = 30) and the unmonitored group (n = 38). The results of the clinical baseline characteristics are summarized in Table 1. There were no differences in age, sex, BMI, tumour location, pathological grade, TV, preoperative KPS score or ECOG grade. Thus, the preoperative clinical characteristics of the two groups were well matched.

**Table 1:** Demographical characteristics and clinical data of the patients

Variables (n = 68)	Monitored group (n = 30)	Unmonitored group (n = 38)	P value
Age (years)	58.8 ± 12.5	58.0 ± 12.2	0.791
Gender			0.625
Male, n (%)	16 (53.3)	18 (47.4)	
Female, n (%)	14 (46.7)	20 (52.6)	
BMI (kg/m^2^)	27.3 ± 5.1	28.0 ± 5.0	0.622
Dominent hemisphere			0.329
Left, n (%)	17 (56.7)	17 (44.7)	
Right, n (%)	13 (43.3)	21 (55.3)	
Glioma WHO grade			0.990
Grade II, n (%)	9 (30.0)	11 (28.9)	
Grade III, n (%)	12 (40.0)	15 (39.5)	
Grade IV, n (%)	9 (30.0)	12 (31.6)	
Pre-operative KPS score	80.0 (70.0–80.0)	80.0 (70.0–80.0)	0.750
Pre-operative ECOG grade			0.602
Grade I, n (%)	20 (66.7)	23 (60.5)	
Grade II, n (%)	10 (33.3)	15 (39.5)	
Tumor volume			
TV (cm^3^)	32.0 (23.6–48.0)	30.5 (25.3–41.4)	0.961
RV (cm^3^)	1.4 (0.6–2.4)	1.7 (0.7–2.5)	0.688
EOR (%)	94.9 (92.5–98.2)	95.1 (92.8–97.5)	0.911
Operative blood loss (ml)	285.0 (260.0–300.0)	295.0 (260.0–310.0)	0.303
Duration of surgery (minutes)	333.5 (316.0–349.8)	322.5 (302.8–540.5)	0.201
Length of hospital stay (days)	25.0 (21.8–27.3)	27.5 (23.8–28.3)	0.056

BMI, Body Mass Index; TV, Preoperative tumor volume; RV, Post-operative residual tumor volume; EOR, Extent of resection; KPS, Karnofsky performance Score; ECOG, Eastern Cooperative Oncology Group grade

Student t test, mean ± standard, *P < 0.05, **P < 0.01

Chi-square test, n (%), *P < 0.05, **P < 0.01

Mann–Whitney U test, Median (Q1–Q3), *P < 0.05, **P < 0.01

### Surgical indicators of postoperative outcomes

To reveal whether IONM had an effect on the extent of surgical resection, postoperative residual tumour volume (RV) and extent of resection (EOR) were compared, as shown in Table 1. The median RV was 1.4 cm^3^ (0.6–2.4 cm^3^) vs. 1.7 cm^3^ (0.7–2.5 cm^3^), and the median EOR was 94.9% (92.5–98.2%) vs. 95.1% (92.8–97.5%) in the monitored and unmonitored groups, respectively. The Mann‒Whitney U test did not show any significant differences in RV and EOR.

In addition, the surgical information, including intraoperative bleeding volume, duration of surgery and length of hospital stay, is also compared in Table 1. Overall, the surgical treatment indicators and the hospital stay of the monitored group seemed to be better than those of the unmonitored group, although the difference was not statistically significant.

### Functional outcome analysis

To probe the functional outcome relevance of IONM, we used the KPS score and ECOG grade to compare the benefits of patients at two weeks postoperation and three months follow-up. As shown in Table 2, neither the postoperative KPS score nor the ECOG grade were significantly different between the two groups. However, we investigated the functional outcome at the three-month follow-up, and the median KPS scores were 80.0 (70.0–80.0) vs. 70.0 (70.0–80.0), respectively. The KPS score of the monitored group was significantly higher than that of the unmonitored group, and the same conclusion could be drawn by the comparison of ECOG grades.

**Table 2:** The neurological function status at two post-operation and three-month follow-up

Variables (n = 68)	Two weeks Post-operation			Three months follow-up		
	Monitored Group(n = 30)	Unmonitored group(n = 38)	P value	Monitored group(n = 30)	Unmonitored group(n = 38)	P value
KPS Score	70.0 (70.0–80.0)	70.0 (67.5–80.0)	0.531	80.0 (70.0–80.0)	70.0 (70.0–80.0)	0.001**
ECOG grade			0.177			0.005**
Grade I, n(%)	12(40.0)	15(39.5)		21(70.0)	13(34.2)	
Grade II, n(%)	18(60.0)	19(50.0)		9(30.0)	19(50.5)	
Grade III, n(%)	0(0)	4(10.5)		0(0)	6(15.8)	
Function status			0.695			0.014**
Group A	18(60.0)	21(55.3)		23(76.7)	18(47.4)	
Group B	12(40.0)	17(44.7)		7(23.3)	20(52.6)	

Chi-square test, n(%), *P < 0.05, **P < 0.01

Mann–Whitney U test, Median(Q1-Q3), *P < 0.05, **P < 0.01

Group A, Patients with function improved or unchanged

Group B,  Patients with function deteriorated

**Fig. 1:**
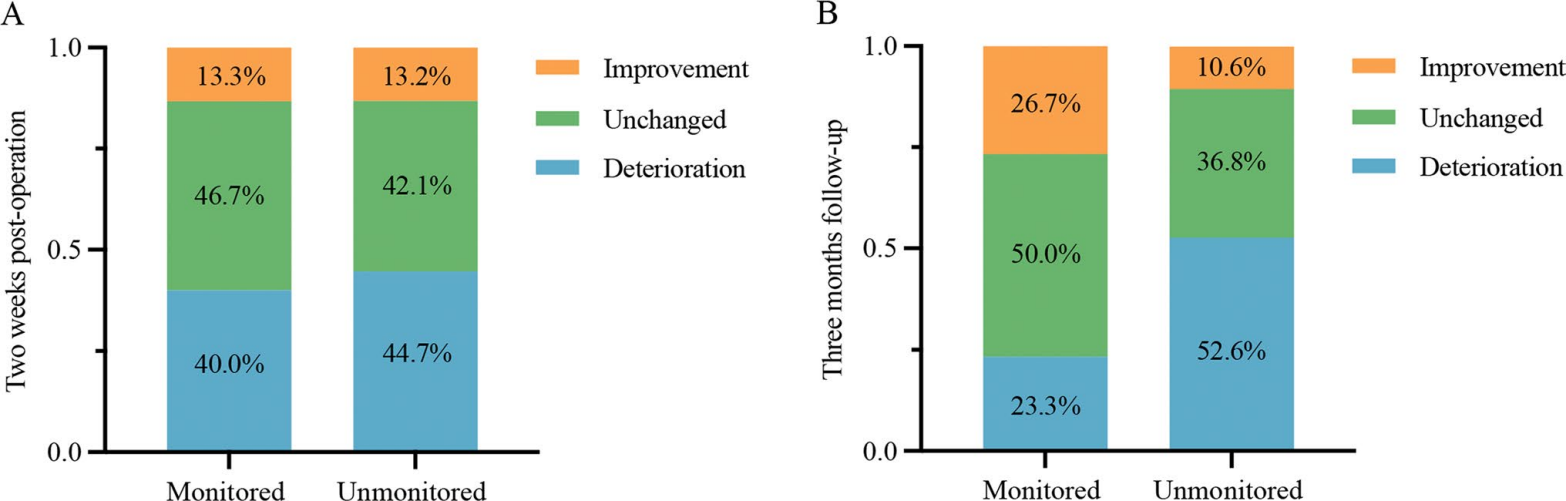
The incidence rates of the different neurological function status. **A** At two weeks post-operation, there was no significant difference between monitored group and unmonitored group (P > 0.05). **B** At 3 month follow-up, the percentage of patients who function deteriorated in monitored group was significantly lower than that in unmonitored group (P < 0.05)

Then, we combined the KPS score with the ECOG grade to evaluate the neurological function change at different periods. All patients were defined as having clinical improvement, unchanged or deterioration, and the results are displayed in Fig. 1. At two weeks postoperation, there was no significant difference between the monitored group and the unmonitored group in function status (Table 2). However, comparing the results at the three-month follow-up, the percentage of patients who had deteriorated function in the monitored group was significantly lower than that in the unmonitored group (23.3% vs. 52.6%). Therefore, IONM failed to benefit short-term neurological function, whereas long-term status was significantly improved.

Logistic regression analysis was conducted to assess factors of the long-term neurological function status among all patients in Supplementary Table 1. We found that IONM (OR = 0.23, 95% CI 0.07–0.70) was a significant factor associated with the protection of long-term neurological function.

### Survival analysis

**Fig. 2:**
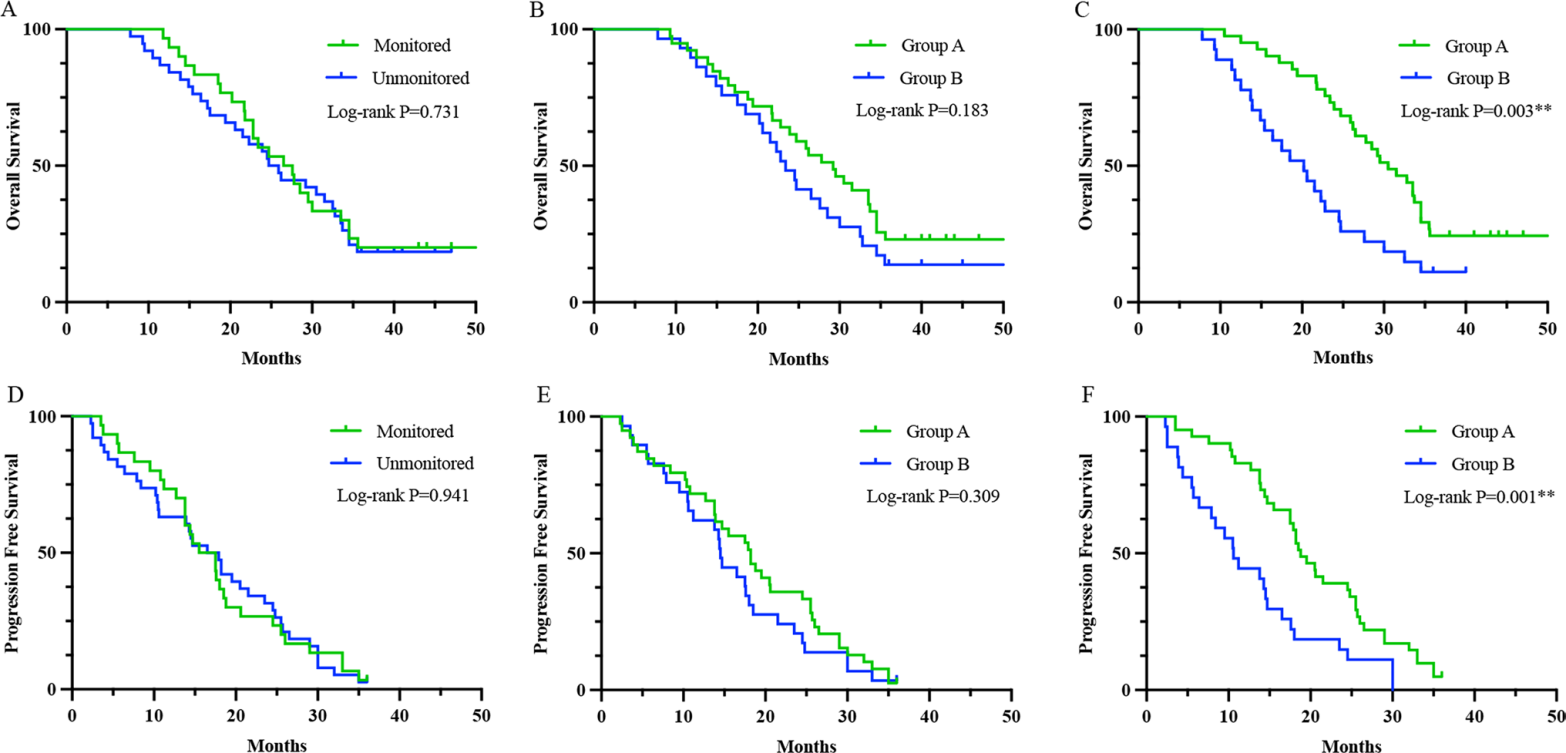
The Kaplan–Meier curves were used to show survival outcomes. **A**, **D** Application of IONM was not associated with favorable OS and PFS (P > 0.05). **B**, **E** The postoperative neurological status did not affect the OS and PFS (P > 0.05). **C**, **F** The long-term neurological deterioration was associated with worsened OS and PFS (P < 0.05)

The correlation between IONM and the prognosis of patients is shown in Fig. 2a, d. Kaplan–Meier survival curves with the log-rank test showed no statistically significant difference in OS and PFS. Then, we evaluated the effect of neurological function status on patient survival. The functional difference at two weeks postoperatively did not affect OS or PFS (Fig. 2b, e). However, at the three-month follow-up, the median OS time of the two groups was 30.5 months vs. 20.2 months, and the median PFS was 18.8 months vs. 10.6 months (Fig. 2c, f). The differences were statistically significant. In summary, long-term neurological deterioration indicated an obviously worsened prognosis.

Clinical characteristics combined with the neurological function status for the prediction of OS were further investigated by univariate analysis with the Cox regression model. In univariate analysis, glioma WHO grade, RV, EOR and long-term functional status were significantly associated with OS (Table 3). The significant factors in univariate Cox regression analyses were further subjected to multivariate Cox regression analysis, in which glioma WHO grade, EOR, and long-term functional status were verified to be independent prognostic factors for OS.

**Table 3:** Univariate and multivariate survival analyses of OS in eloquent area glioma patients

	Univariate			Multivariable		
	HR	95% CI	P value	HR	95% CI	P value
Age (< 60/ ≥ 60 years)	0.79	0.46–1.34	0.373			
Gender (Male/Female)	0.69	0.41–1.18	0.175			
BMI (< 28/ ≥ 28 kg/m^2^)	0.66	0.39–1.14	0.135			
Dominent hemisphere (Left/Right)	0.90	0.53–1.53	0.690			
Glioma WHO grade (Low/High grade)	4.98	2.50–9.91	< 0.001*	4.95	2.28–10.73	< 0.001*
TV (< 30/ ≥ 30 cm^3^)	1.14	0.67–1.94	0.634			
RV (< 1.5/ ≥ 1.5 cm^3^)	2.09	1.21–3.58	0.008*	1.81	0.86–3.80	0.119
EOR (< 95/ ≥ 95%)	0.26	0.15–0.47	< 0.001*	0.43	0.19–0.97	0.041*
Operative blood loss (< 290/ ≥ 290 ml)	0.90	0.53–1.53	0.692			
Duration of surgery (< 330/ ≥ 330 min)	1.49	0.87–2.55	0.143			
Length of hospital stay (< 25/ ≥ 25 days)	1.09	0.63–1.88	0.755			
Pre-operative KPS Score (< 80/ ≥ 80)	0.77	0.45–1.32	0.340			
Post-operative KPS Score (< 80/ ≥ 80)	0.60	0.34–1.05	0.074			
Follow-up KPS score (< 80/ ≥ 80)	0.61	0.36–1.03	0.066			
Short-term function status (Group A/Group B)	1.43	0.84–2.44	0.189			
Long-term function status (Group A/Group B)	2.23	1.30–3.82	0.004*	2.14	1.23–3.71	0.007*
IONM (Yes/No)	0.91	0.54–1.55	0.734			

Cox proportional hazard regression,*P < 0.05, **P < 0.01

IONM, Intraoperative neurophysiological monitoring; CI, Confidential interval; HR, Hazard rate

Group A, Patients with function improved or unchanged

Group B, Patients with function deteriorated

**Fig. 3:**
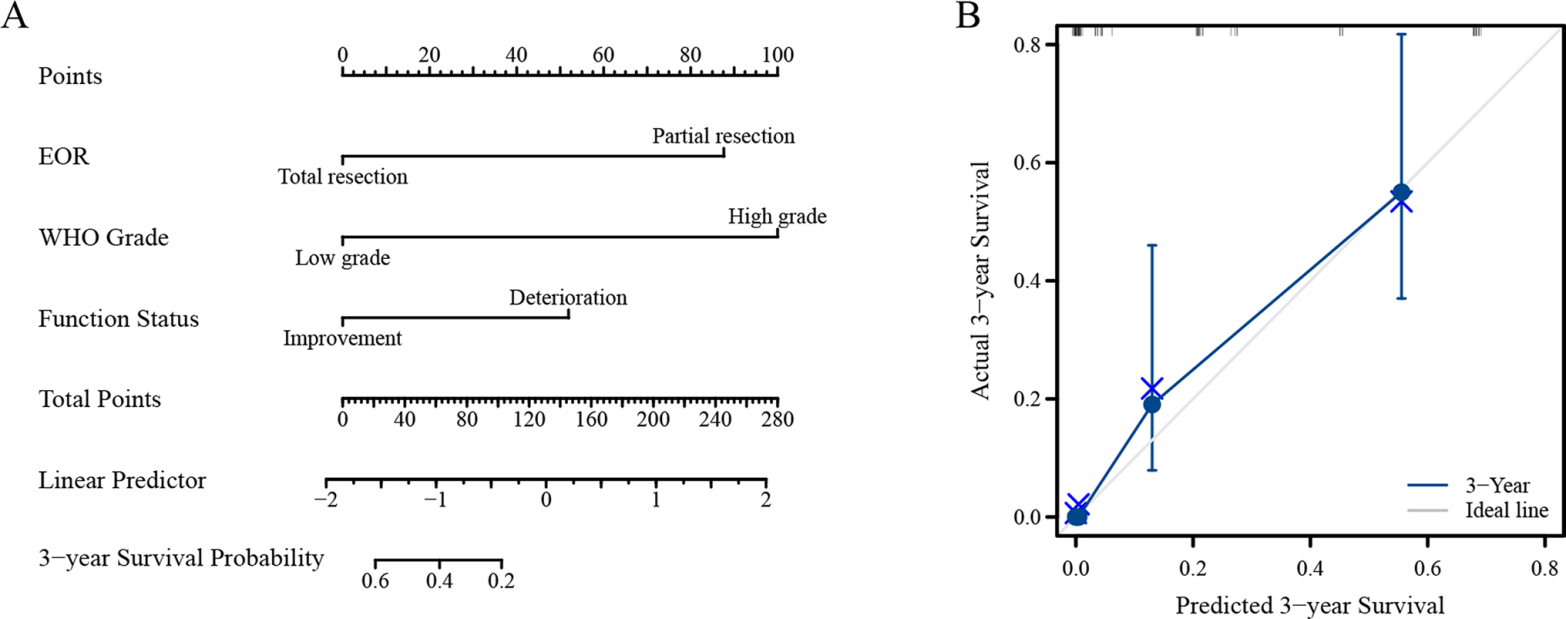
The nomogram was established to predict the 3-year survival rates. **A** The long-term function status, glioma malignant degree and EOR were verified to be independent prognostic factors for OS. **B** Calibration plots of nomograms for predicting OS in patients with eloquent area glioma.The x-axis is nomogram-predicted probability of survival and the y-axis is actual survival. The reference line is 45° and indicates perfect calibration

To predict the OS of patients with eloquent area glioma, a prognostic nomogram was established through Cox regression model analysis. which could predict the probability 3-year survival rates (Fig. 3a). Harrell’s c-index was 0.761 (95% CI 0.734–0.788), and the calibration curves showed good agreement between the prediction by the nomogram and the actual observation (Fig. 3b).

### Predictive value of IONM

**Fig. 4:**
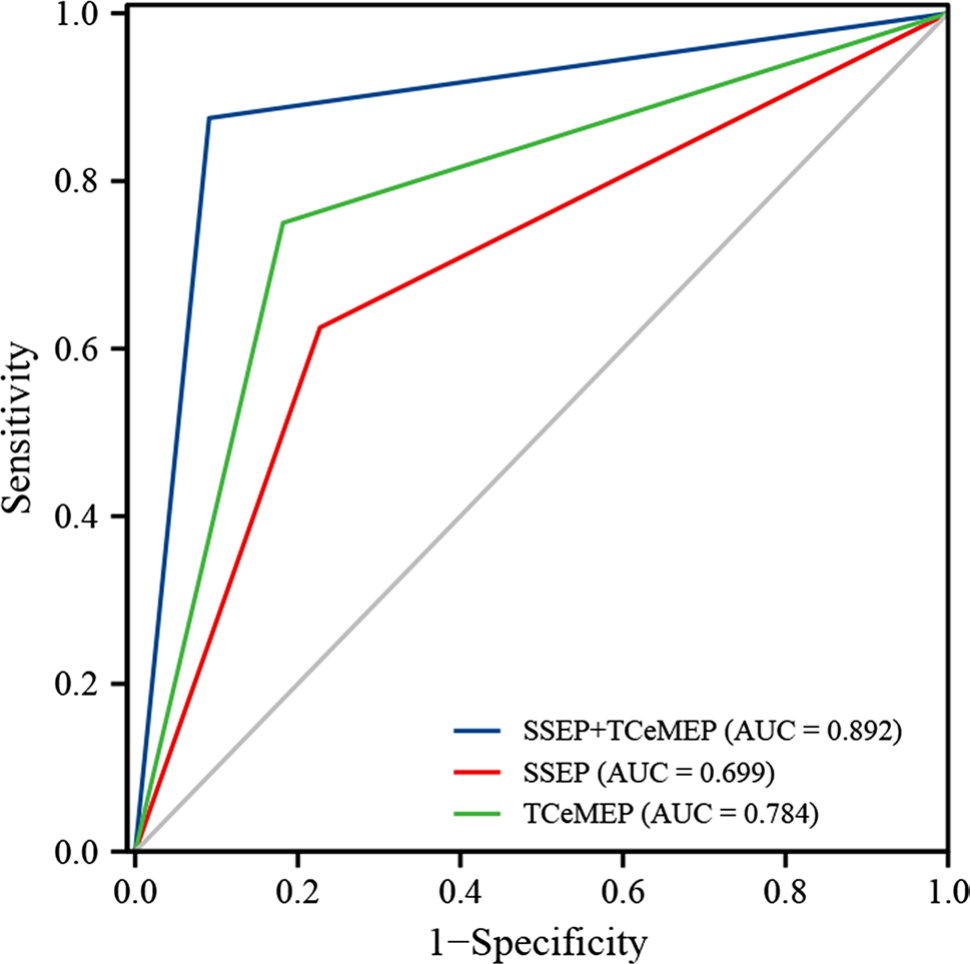
ROC curves for the IONM evoked potential (SSEP and TCeMEP). The AUC of IONM evoked potential (both SSEP and TCeMEP) was 0.892, which is significantly reliable in detecting neurologic function status after surgery (P < 0.05)

Multimodal IONM, including SSEP and TCeMEP, was utilized for all eloquent area glioma patients. The sensitivity, specificity, negative predictive value (NPV), positive predictive value (PPV), positive likelihood ratio (LR +) and negative likelihood ratio (LR −) analyses of each evoked potential are shown in Table 4. The evoked potentials (SSEP and TCeMEP) significantly predicted postoperative permanent functional deficits, with a sensitivity of 0.875 and a specificity of 0.909. In this ROC curve analysis, the area under the curve (AUC) for the SSEP and TCeMEP curves was 0.892, which indicated that the ROC was statistically significant and that the results had high accuracy (Fig. 4).

**Table 4:** Sensitivity, specificity, PPV, NPV, positive and negative likelihood ratios, AUC, and statistical analysis of IONM

	SSEP and TCeMEP	SSEP	TCeMEP
True negative	20	17	18
True positive	7	5	6
False negative	1	3	2
False positive	2	5	4
Sensitivity	87.5%	62.5%	75.0%
Specificity	90.9%	77.3%	81.8%
PPV	77.8%	50.0%	60.0%
NPV	95.2%	85.0%	90.0%
LR +	9.625	2.750	4.125
LR-	0.138	0.485	0.306
AUC	0.892	0.699	0.784
95%CI	0.755–1.000	0.498–0.899	0.604–0.964
P value	0.032*	0.123	0.200

PPV, Positive predictive value; NPV, Negative predictive valve; LR +, Likelihood ratio Positive; LR−, Likelihood ratio negative; AUC, The area under curve

^*^P < 0.05, **P < 0.01

## Discussion

Gliomas that infiltrate eloquent areas and subcortical fibres can damage the corresponding cortical or subcortical structures, leading to neurological dysfunction [17]. Many studies have shown that EOR is an important prognostic factor for glioma patients [18], and gross total resection (GTR) can prolong survival and delay recurrence [19, 20]. Grabowski et al. [21] showed that either RV ≤ 2 cm^3^ or EOR ≥ 98% was beneficial for survival outcomes. Chaichana et al. [22] stated that RV and EOR with the greatest reduction in the risk of death were < 2 cm^3^ and > 95%, respectively. Therefore, the widely accepted surgical strategy is to achieve maximum safe excision, which reflects the need to prolong survival and improve neurological function [4]. Especially for high-grade glioma patients with a short life expectancy, neurological dysfunction after surgery significantly affects quality of life [5]. For this reason, multiple functional imaging and monitoring techniques would help with preoperative and intraoperative surgical decisions to identify and protect cerebral eloquent areas.

Preoperative examinations, including f-MRI and DTI, are commonly used to investigate anatomical structure and functional localization. Various intraoperative technologies, such as the neuronavigation system, intraoperative MRI (io-MRI), 5-ALA-induced ppIX fluorescence imaging, and ultrasonic systems, have been demonstrated as effective techniques in detecting tumour boundaries [23]. However, for accurately mapping cerebral eloquent areas, image-guided technology alone is not enough. Barone et al. [24] showed that io-MRI, 5-ALA or DTI-neuronavigation can increase the proportion of GTR on postoperative MRI, but maximizing the EOR may lead to more frequent adverse events. Because of the displacement or remodelling of the cerebral area caused by glioma, IONM has become an indispensable tool for maintaining neurological function in surgery.

Currently, the value of IONM in neurosurgical surgery has been extensively studied and validated. Based on a long-term follow-up of 856 samples of patients, Zhang et al. [25] reported that IONM was a favourable factor of OS and late neurologic function in glioma surgery. Pan et al. [26] stated that IONM could be beneficial for postoperative functional preservation but was not significantly associated with EOR, OS or PFS. In this study, we revealed the resection impact of IONM in eloquent areas of gliomas. The median EOR tended to be 95% (94.9% and 95.1%), and both median RVs were less than 2 cm^3^ (1.4 cm^3^ and 1.7 cm^3^), which obviously reached the effective threshold of resection. Other surgical information, including intraoperative bleeding volume, duration of surgery and length of hospital stay, was not significantly different between the monitored and unmonitored groups, but the above data are comparable to the international average. This favourable outcome may be attributed to the fact that multiple functional image-guided techniques and the long-term accumulation of experience in the surgeon.

Then, we probed the contribution of IONM to functional outcomes. In the monitored group, 12 patients (40.0%) had neurologic deficits at two weeks postoperation, of whom 5 patients (16.7%) were restored to the preoperative level at the three-month follow-up. For the unmonitored group, 17 patients (44.7%) had permanent neurologic deficits, and 3 patients (7.9%) had delayed neurological deterioration. The valuable finding is that the rates of neurologic deterioration at two weeks postoperation were not significantly different, while the rates of neurologic deterioration at the three-month follow-up were significantly lower in the monitored group than in the unmonitored group. We inferred that the advantage of long-term functional status demonstrates the contribution of IONM to the protection of nerve nuclei and nerve conduction tracts, thus providing a condition for neurological function recovery of early neurologic deficits. In the current monitoring methods, phase inversion of SSEPs acts as one of the valid parameters for the localization of the central sulcus throughout the tumour resections [27]. DCS and DsCS are even the most reliable methods of functional brain mapping and are considered the “gold standard” for real-time detection of cerebral eloquent areas in glioma resection [27, 28]. These modalities were applied as needed in glioma resection to achieve brain mapping. In 21 patients (66.7%), phase inversion of SSEPs was used to locate the central trench with 100% accuracy. DCS and DsCS were performed to ensure the boundary of resection in 19 patients (63.3%), and the alarm signal occurred in 11 patients (36.7%). The signal of 3 patients (10.0%) was not recovered at the end of resection and led to permanent neurologic deficits. Notably, as DCS and DsCS may cause intraoperative seizures, ECoG should be performed for real-time monitoring [29]]. In our study, intraoperative seizures were detected in 3 patients (7.9%), and the stimulation was stopped immediately. Meanwhile, cold saline irrigation and propofol bolus infusion were used for effective control.

Over the past few decades, the eloquent location of gliomas has been regarded as a hazard factor in predicting poor prognosis [30], but an increasing number of studies have shown that the eloquent location should not be considered a determinant affecting prognosis due to the progression of various techniques for neurological protection [25]. Our study found that the OS and PFS of patients with and without IONM were not significantly different. Nevertheless, we further found that postoperative neurological function status was correlated with survival outcome. The Kaplan‒Meier survival analysis revealed no statistical differentiation for OS and PFS in postoperative short-term functional status, while significantly longer survival was observed in patients with long-term functional improvement. In addition, using the Cox regression analysis and nomogram model, the long-term functional status combined with WHO grade and EOR had good predictive capability for 3-year survival rates when they incorporated integrated information. Therefore, we conclude that the improved OS and PFS in the long-term function improvement group may come from the application of IONM, in light of the fact that neurologic function deterioration will negatively influence quality of life after surgery and subsequently impair survival outcomes.

At present, the challenges of IONM are still the interpretation of intraoperative warning signals and the correct judgement of false-negatives and false-positives [31]. Thus, our study reviewed IONM evoked potential (SSEP and TCeMEP) changes and evaluated which combination of these modalities was more precise in identifying postoperative sensory and/or motor deficits. The alarm of SSEPs could indicate an insult to the sensory pathway and lead to a postoperative sensory deficit, but based on our data, the sensitivity of SSEP changes was unsatisfactory (62.5%). However, MEP change had high sensitivity (75.0%) and specificity (81.8%) for predicting postoperative motor deficits. The ROC curve results indicated that TCeMEP combined with SSEP could be significantly reliable in detecting neurologic function status (AUC = 0.892, P < 0.05).

The limitations of this study should be acknowledged here. First, this was a retrospective study with all the inherent limitations, and the period of clinical data collection was different between the monitored group and the unmonitored group. Even in future prospective studies, it is almost impossible to completely eliminate this bias due to ethical limitations. Second, the sample size of our study cohort was relatively small (n = 68 patients); thus, the statistical power of the multivariate analysis may have been reduced. Finally, the follow-up duration of the study may not be long enough, especially for LGG patients. Therefore, we will further continuous the follow-up time and include a second independent centre to expand the sample size in the future.

## Conclusions

The findings of this retrospective cohort study indicate that the application of IONM are superior in improving the long-term neurological function in patients with eloquent area glioma. Based on the postoperative function status, EOR and glioma malignant degree, a nomogram may be a more practical model for evaluating prognosis. The recording of IONM evoked potential (SSEP and TCeMEP) could accurately detect postoperative function deficits for gliomas patients involving eloquent areas.

### Supplementary Information

**Additional file 1. **

**Additional file 2. **

## Data Availability

The original data presented in the study are included in the article/supplementary Table, and further inquiries can be directed to the corresponding author.
