# Supplementary material for: Application value of intraoperative electrophysiological monitoring in cerebral eloquent area glioma surgery: a retrospective cohort study
Source: Discov Oncol. 2024 Apr 13;15:118. doi: 10.1007/s12672-024-00975-5 (PMC11016029; doi:10.1007/s12672-024-00975-5)
Supplement: Supplementary file 1 — Additional file 1. [file 12672_2024_975_MOESM1_ESM.docx]

**Supplementary Table 1.** Univariate and multivariate logistic regression analysis of factors associated with long-term neurological function status.

|  | Univariate | | |  |  |  | Multivariable | | |
| --- | --- | --- | --- | --- | --- | --- | --- | --- | --- |
|  | Group A | Group B | 0R | 95%CI | P value |  | OR | 95%CI | P value |
| Age (years) |  |  |  |  |  |  |  |  |  |
| <60 | 21 | 12 |  |  |  |  |  |  |  |
| ≥60 | 21 | 14 | 1.31 | 0.50-3.48 | 0.585 |  |  |  |  |
| Gender |  |  |  |  |  |  |  |  |  |
| Male | 23 | 11 |  |  |  |  |  |  |  |
| Female | 19 | 15 | 0.69 | 0.26-1.83 | 0.458 |  |  |  |  |
| BMI(kg/m^2^) | | | |  |  |  |  |  |  |
| <28 | 20 | 16 |  |  |  |  |  |  |  |
| ≥28 | 22 | 10 | 0.84 | 0.32-2.23 | 0.726 |  |  |  |  |
| Dominent hemisphere | | | |  |  |  |  |  |  |
| Left | 22 | 12 |  |  |  |  |  |  |  |
| Right | 20 | 14 | 1.13 | 0.43-2.99 | 0.804 |  |  |  |  |
| Glioma WHO grade | | | |  |  |  |  |  |  |
| Low grade | 16 | 5 |  |  |  |  |  |  |  |
| High grade | 26 | 21 | 2.82 | 0.89-8.95 | 0.079 |  |  |  |  |
| TV(cm^3^) | | | |  |  |  |  |  |  |
| <30 | 20 | 12 |  |  |  |  |  |  |  |
| ≥30 | 22 | 14 | 0.93 | 0.35-2.46 | 0.884 |  |  |  |  |
| RV(cm^3^) | | | |  |  |  |  |  |  |
| <1.5 | 20 | 13 |  |  |  |  |  |  |  |
| ≥1.5 | 22 | 13 | 1.13 | 0.43-2.99 | 0.804 |  |  |  |  |
| EOR(%) | | | |  |  |  |  |  |  |
| <95 | 20 | 14 |  |  |  |  |  |  |  |
| ≥95 | 22 | 12 | 0.73 | 0.27-1.93 | 0.521 |  |  |  |  |
| Operative blood loss(ml) | | | |  |  |  |  |  |  |
| <290 | 20 | 10 |  |  |  |  |  |  |  |
| ≥290 | 22 | 16 | 2.10 | 0.77-5.75 | 0.149 |  |  |  |  |
| Duration of surgery(minutes) | | |  |  |  |  |  |  |  |
| <330 | 22 | 14 |  |  |  |  |  |  |  |
| ≥330 | 20 | 12 | 0.59 | 0.22-1.59 | 0.299 |  |  |  |  |
| Length of hospital stay(days) | | |  |  |  |  |  |  |  |
| <25 | 18 | 8 |  |  |  |  |  |  |  |
| ≥25 | 24 | 18 | 1.86 | 0.66-5.21 | 0.239 |  |  |  |  |
| Pre-operative KPS Score | | |  |  |  |  |  |  |  |
| <80 | 21 | 5 |  |  |  |  |  |  |  |
| ≥80 | 21 | 21 | 3.02 | 1.01-9.04 | 0.048* |  | 3.78 | 1.17-12.24 | 0.027* |
| IONM | | |  |  |  |  |  |  |  |
| Yes | 23 | 7 |  |  |  |  |  |  |  |
| No | 19 | 19 | 0.27 | 0.10-0.79 | 0.017* |  | 0.23 | 0.07-0.70 | 0.010* |

*P<0.05, **P<0.01

Group A= Patients with function improved or unchanged, Group B=Patients with function deteriorated, BMI=Body Mass Index, TV=Preoperative tumor volume, RV=Post-operative residual tumor volume, EOR=Extent of resection, KPS=Karnofsky performance Score, IONM=Intraoperative neurophysiological monitoring, CI=Confidential interval, OR=Odds ratio.
